# The Standard Dictionary of Funk & Wagnalls

**Published:** 1894-11

**Authors:** 


### BOOK NOTICES.

THE STANDARD DICTIONARY, of Funk & Wagnalls, was reviewed in the pages of THE HOMŒOPATHIC PHYSICIAN, in April last, at page 122. From time to time since that issue of the journal we have called attention to particular merits as they occurred to us. We once more bring it to the notice of our readers, with the announcement that the second volume is finished and will soon be ready for delivery. We now append the special card of the publishers to the editor, as it is more comprehensive.

After five years of labor, with the help of 247 editors and the enormous expenditure of nearly one million dollars, the Funk & Wagnalls Company announce that the last page of the second, the concluding volume of the new *Standard Dictionary*, is now in type. This volume will be ready for delivery in November.

The hearty reception extended the Funk & Wagnalls *Standard Dictionary* by the literary public in England is one of the literary surprises of the past year. The eminent English novelist, A. Conan Doyle, now lecturing in this country, recently wrote from London to the publishers as follows:

LONDON, 12 Tennison Road, South Norwood, August 20th, 1894.

GENTLEMEN:—I wrote once before to commend your dictionary, but I feel bound to do so again after a longer experience with it. It has become quite a joke with us that we cannot trip it up. We have several times been sure that we would, but have always failed. Within the last week I have had occasion to turn it up for "gyp," "coffle," and "coshering," always successfully. Is the second volume purchasable?

Yours faithfully,

A. CONAN DOYLE.

Hon. Justin McCarthy, the historian, and member of the House of Commons, London, Eng., recently wrote:

"I refer to it (the *Standard Dictionary*) every day—never once without feeling that it has given me a helping hand in my studies and in my writings. I regard it as a monumental work—a work, thus far, perfect of its kind, and for its purpose destined to be a conclusive authority to the English-speaking peoples, and to other peoples as well, for many a generation."

The sales of the new *Standard Dictionary* are phenomenal. The publishers have a mathematician who has figured out that if the copies required to fill the advance orders were laid one on top of the other the stack would be over three miles high, and laid end to end would make a path over fifteen miles in length.

A general agent in Michigan startled the publishers of the new *Standard Dictionary* by an order for two car-loads—43,000 pounds—of dictionaries, to be sent as soon as Volume II is ready.

